# Immune infiltration landscape and potential drug-targeted implications for hepatocellular carcinoma with ‘progression/hyper-progression’ recurrence

**DOI:** 10.1080/07853890.2025.2456113

**Published:** 2025-01-27

**Authors:** Jing-Xuan Xu, Yue-Xiang Su, Yuan-Yuan Chen, Yi-Yue Huang, Zu-Shun Chen, Yu-Chong Peng, Lu-Nan Qi

**Affiliations:** ^a^ Department of Hepatobiliary Surgery, Guangxi Medical University Cancer Hospital, Nanning, China; ^b^ Key Laboratory of Early Prevention and Treatment for Regional High Frequency Tumour, Ministry of Education, Nanning, China; ^c^ Department of Ultrasound, First Affiliated Hospital of Guangxi Medical University, Nanning, China; ^d^ Department of General Surgery, Chongqing Hospital of Traditional Chinese Medicine, Chongqing, China; ^e^ Guangxi Liver Cancer Diagnosis and Treatment Engineering and Technology Research Center, Nanning, China

**Keywords:** Hepatocellular carcinoma, progression/hyper-progression, immunophenotyping, immune intermediate state, extracellular matrix, Huaier granules, lenvatinib

## Abstract

**Background and aims:**

Hepatocellular carcinoma (HCC) recurrence was previously characterized into four types, and patients with progression/hyper-progression recurrence (type III–IV) have an extremely poor prognosis. However, the immune background of resectable HCC, particularly in patients who experience recurrence, remains underexplored. Therefore, this study aimed to describe the immune landscape of resectable HCC, especially postoperative type III–IV recurrent HCC, and explore potential immune-targeted anti-relapse strategies for treated populations.

**Methods:**

The differences in gene expression in patients with recurrent HCC (type I–II (solitary or multi-intrahepatic oligo recurrence) vs. type III–IV) were investigated using bulk sequencing. Multiple immune infiltration methods (single-sample gene set enrichment analysis (GSEA), Microenvironment Cell Populations-counter and ESTIMATE) were used, and patients were divided into four groups to identify four distinct immune subtypes: immune-enrichment/matrix-poor (IE1), immune-enrichment/matrix-rich (IE2), immune intermediate/matrix-rich (ITM) and immune desert/matrix-poor (ID). Co-expression and protein interaction analyses were used to identify characteristic genes in ITM closely associated with type III–IV recurrence, which was matched with drug targets for Huaier granules (HG) and lenvatinib. Virtual docking was used to identify potential therapeutic targets, and the results were verified using single-nuclei RNA sequencing and histological analysis.

**Results:**

ITM was closely related to type III–IV recurrence and exhibited immunotherapy potential. The potential efficacy of inhibiting CCNA2, VEGFA, CXCL8, PLK2, TIMP1, ITGB2, ALDOA, ANXA5 and CSK in ITM reversal was determined. Molecular docking demonstrated that the proteins of these genes could bind to HG or lenvatinib. The immunohistochemical findings demonstrated differential VEGFA (*p* < .01) and PLK2 (*p* < .001) expression in ITM type and ID in type III–IV recurrent HCC.

**Conclusions:**

Three primary immunotypes of resectable HCC (IE2, ITM and ID) were identified, and HG and lenvatinib could potentially overcome immune checkpoint blockade (ICB) resistance in ITM patients with HCC, particularly those classified as type III–IV.

## Introduction

Hepatocellular carcinoma (HCC) is a prevalent subtype of liver cancer and is the third leading cause of cancer-related mortality [1]. Surgery is the primary and most effective treatment for HCC; however, HCC recurrence and metastasis remain substantial challenges [2]. A novel ‘four-type recurrent HCC classification’ was previously introduced, emphasizing the extremely poor prognosis associated with progression or hyper-progression recurrence (type III–IV recurrence) [3]. Postoperative anti-recurrence adjuvant therapy, including immune-targeted therapy, is crucial to improve the short- or long-term prognosis of patients.

Although immunotherapies, particularly immune checkpoint blockade (ICB), have improved outcomes in many cancer types, most patients with HCC fail to exhibit a clinical response [4–6]. Various combination therapies, including trans-arterial chemoembolization with ICB, are currently being investigated; however, these approaches face substantial challenges owing to the high incidence of adverse events and a lack of clarity in identifying the patient populations most likely to benefit [7]. These limitations may be attributed to the heterogeneity of recurrence patterns and variations in the immune microenvironment and biological characteristics of primary tumours. Previous studies on the tumour microenvironment (TME) of HCC have predominantly focused on patients with overall liver cancer [8–11], especially those with unresectable cases [12,13]. Only a few studies have specifically examined the TME of multifocal primary tumours [14,15]. However, the immune background of resectable HCC, especially in patients with recurrent outcomes, has not been explored. Therefore, further research is necessary to identify the benefits of ICB treatment and select the best immune-targeted drugs or combination treatment regimens.

The TME plays a pivotal role in immune tolerance and escape through multiple mechanisms [16–18]. The TME comprises tumour cells, immune cells, the extracellular matrix (ECM) – an intricate cytokine environment – and other components that create an extensive network of interactions [18–20]. Immune cell infiltration and stromal enrichment are the key parameters evaluated in pan-cancer immunophenotyping studies [21,22]. However, the understanding of complex cellular interactions in the TME of HCC is incomplete. A deeper understanding of these interactions holds the potential for developing innovative immunotherapies for HCC.

The immune intermediate state, lying between immune-inflamed and immune-deserted phenotypes, presents a region where immune cells and matrix components (including tumour-associated stromal cells and the ECM) are highly intertwined [22], posing considerable obstacles to HCC immunotherapy [21]. The stromal cell compartment consists of many cell types, including endothelial cells, fibroblasts and mesenchymal stromal cells [23–25]. Cancer-associated fibroblasts (CAFs) [26,27], endothelial cells and angiogenic signals [28–30] from stromal cells influence therapeutic outcomes. Moreover, stromal cells and secreted cytokines within the TME are genetically stable, making them promising therapeutic targets with a reduced risk of resistance. Therefore, matrix components and angiogenic factors warrant further investigation.

When analysing the differences in gene expression between type III–IV and I–II recurrences, patients with type III–IV recurrence exhibited pronounced immunosuppressive characteristics, highlighting the need for in-depth exploration of the background of primary tumour immunity in patients with each type of recurrence. Therefore, this study aimed to delineate and classify the primary tumour immune landscape of patients with four subtype HCC relapse, focusing on identifying inhibitor targets in immune intermediate-types closely related to types III–IV HCC. Molecular docking of potential targets with lenvatinib – approved as a first-line immunotherapy for unresectable HCC [31] – as well as with the active ingredients of the emerging traditional Chinese medicine, Huaier granules (HG), was performed to provide a valuable decision-making basis for the treatment of HCC after surgery.

## Materials and methods

### Patients and samples

All tissue samples and clinical data were collected after ethical review and approval by the ethics committee of Guangxi Medical University Cancer Hospital (approval number: LW2023122). Written informed consent was obtained from all recruited patients. From July 2015 to November 2022, 118 tumour samples were obtained from patients who had had hepatectomy at the Guangxi Medical University Cancer Hospital (Nanning, China) and had been diagnosed with HCC by pathology. All samples and clinical data were retrospectively collected. Among the 118 patients, 68 had type I–II recurrence, while 50 had type III–IV recurrence.

The inclusion criteria were as follows: (1) a definitive pathological diagnosis of HCC in accordance with the World Health Organization’s standards [32]; (2) no history of radiation therapy or transarterial chemoembolization as anticancer treatment; and (3) macroscopic R0 resection, defined as complete macroscopic eradication of the tumour, no detectable intra- or extrahepatic metastatic lesions, and negative resection margins. Patients with other malignancies, those who succumbed to perioperative factors, or those who underwent non-radical resection were excluded.

The criteria for the ‘four-type recurrent HCC classification’ were as follows [3]: type I (solitary intrahepatic oligo recurrence), characterized by a single intrahepatic tumour; type II (multi-intrahepatic oligo recurrence), characterized by intrahepatic recurrence and the number of tumours >1, ≤5; type III (progression recurrence), accompanied by vascular invasion and lung, bone, lymph node or brain metastasis, and no intrahepatic recurrence or the number of intrahepatic tumours were ≤5; and type IV (hyper-progression recurrence), characterized by the number of intrahepatic tumours >5 with or without vascular invasion and extrahepatic metastasis.

### Differential expression and weighted gene co-expression network analysis

A comparative analysis of the gene expression profiles of 118 patients with HCC was performed to elucidate the molecular characteristics of primary tumours in patients with type III–IV recurrence. Differential analysis of gene expression was conducted using the R package limma, and pathway analysis was performed using Gene Ontology and gene set enrichment analysis (GSEA). Weighted gene co-expression network analysis (WGCNA) was performed using the R package, which revealed correlations between phenotypes and gene modules. This analysis was performed using genes that ranked in the first 5000 of the median absolute deviation. After removing abnormal samples, *β* = 6 was selected as the soft threshold (scale-free R2 > 0.9), and a scale-free co-expression network was constructed.

### Immune cell infiltration analysis

Single-sample GSEA (ssGSEA) analysis was performed as previously described to identify knowledge-based functional gene expression signatures (Fges) of tumour, immune, stromal and other cell populations [22]. Subsequently, a manual list of 29 Fges covering cellular and functional properties of the TME was created to describe and classify the immune landscape of transcriptomic genes. These TME attributes were primarily categorized into six groups: (1) angiogenic fibroblasts, (2) antitumor immune infiltrates, (3) pro-tumour immune infiltrates, (4) tumour features, (5) pro-tumour immune infiltrates and (6) other important features. The samples were divided into high and low immune groups based on the primary features of immune inflammation and deserts, as highlighted in previous pan-cancer studies [21,22], with antitumor immune infiltration as the primary feature. The samples were then clustered using fibroblasts as features. Other functional characteristics were provided for demonstration and verification.

The Microenvironment Cell Populations-counter was used to estimate immune cell infiltration. Additionally, ESTIMATE, an expression data-based tumour purity assay algorithm, was used to predict the levels of infiltrating stromal and immune cells.

### Construction of the protein interaction and drug regulatory networks

The STRING database (https://string-db.org/) was used to analyse protein–protein interactions. The resulting protein–protein interaction network was visualized using Cytoscape version 3.9.0. To identify key networks and hub genes, we applied the CytoHubba plugin within Cytoscape, ranking genes based on the Degree. Additionally, drug–target relationships for lenvatinib and HG active components were intersected with the hub genes. Regulatory networks of the matched targets and active drug components were visualized using Cytoscape.

### snRNA-seq data processing

In addition to the bulk sequencing analysis, the expression of potential therapeutic targets was validated using single-cell nucleus sequencing. Single-cell nucleus sequencing data were obtained from primary tumours of 11 randomly selected patients, included in the bulk sequencing, and analysed in R (version 4.2) using the Seurat package. Cells were standard filtered against >5% mitochondria-associated genes or over 7000 gene expressions, resulting in a total of 110,705 cells that were included for further analysis and variable features per sample after normalization. Samples were integrated using Harmony [33], and principal component analysis (PCA), clustering and UMAP dimensional reduction were performed with a resolution of 0.7 after nearest neighbour detection. The novel machine learning method, Ikarus, was used to identify tumour cells, and clusters with over 50% highly variant cells were classified as tumour cells [34]. In addition, the annotation of microenvironmental cells was guided by a pivotal study on the liver cancer microenvironment [35].

### Molecular modelling analysis

The active ingredients of HG and their associated target genes were selected using the HERB database (https://herb.ac.cn/) [36], and the predicted targets of lenvatinib were obtained from the PharmMapper Server (http://www.lilab-ecust.cn/pharmmapper/). The structures of the ligands were downloaded from the PubChem database (https://pubchem.ncbi.nlm.nih.gov/), and the 3D structures of the target proteins were downloaded from the PDB Database (https://www.rcsb.org/). To improve docking accuracy and identify optimal docking poses, molecular docking was performed using the Schrodinger software suite (Schrodinger Suites 2021, New York, NY). Docking scores were calculated using the GlideScore function, and poses with a Glide Score < −5 kcal/mol were considered indicative of strong binding affinities [37].

### Immunohistochemistry

Representative TME markers expressed in the cancer tissues of patients with type III–IV recurrence were further validated using immunohistochemistry with a specialized kit (Pv-6000; Zhongshan Jinqiao, Beijing, China) per the manufacturer’s instructions. The tissue sections were examined under a light microscope. The protein expression of the target gene was assessed by determining the percentage of positive cells and categorized into four grades based on percentage scores: <5% (0), 5–25% (1), 26–50% (2), 51–75% (3) and >75% (4). The staining intensity was divided into four grades (intensity score), as follows: no staining (0), weak staining (1), medium staining (2) and strong staining (3). A total score of 0 was labelled as negative (−), scores ranging from 1 to 3 were labelled as (+), scores of 4–8 were labelled as (+ +) and scores of 9–12 were labelled as (+ + +).

### Statistical analysis

Statistical analyses were performed using R software (R Foundation for Statistical Computing, Vienna, Austria). Two-tailed *p* values <.05 were considered significant.

## Results

### Patients with HCC and type III–IV recurrence exhibit distinct immunosuppressive characteristics

**Figure 1.**
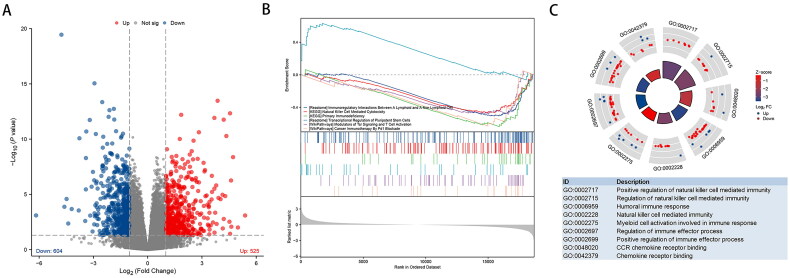
**Differentially expressed gene (DEG) analysis and enrichment analysis of gene expression profiles of type III–IV and type I–II primary tumours.** (A) The volcano plot shows the expression profiles of 558 DEGs in hepatocellular carcinoma (HCC) between type I–II and type III–IV. The high-expressing genes (*n* = 525) are shown in red and the low-expressing genes (*n* = 640) are shown in blue. (B) Gene set enrichment analysis (GSEA) indicates that the immune-related pathways are significantly enriched and (C) Gene Ontology (GO) enrichment analysis also reflects the same trend.

Using ‘I–II type recurrence’ as a reference, 1129 differentially expressed genes associated with type III–IV relapse, including 525 upregulated and 604 downregulated genes, were identified (Figure 1(A)). Enrichment analysis indicated that immune-related pathways were generally downregulated (Table S1, Figure 1(B,C)), whereas the pluripotent stem cell-related pathway was activated (Figure 1(B)).

### Immune infiltration analysis shows the patient’s immune panorama and type III and IV are dominated by immune intermediates and immune deserts

**Figure 2.**
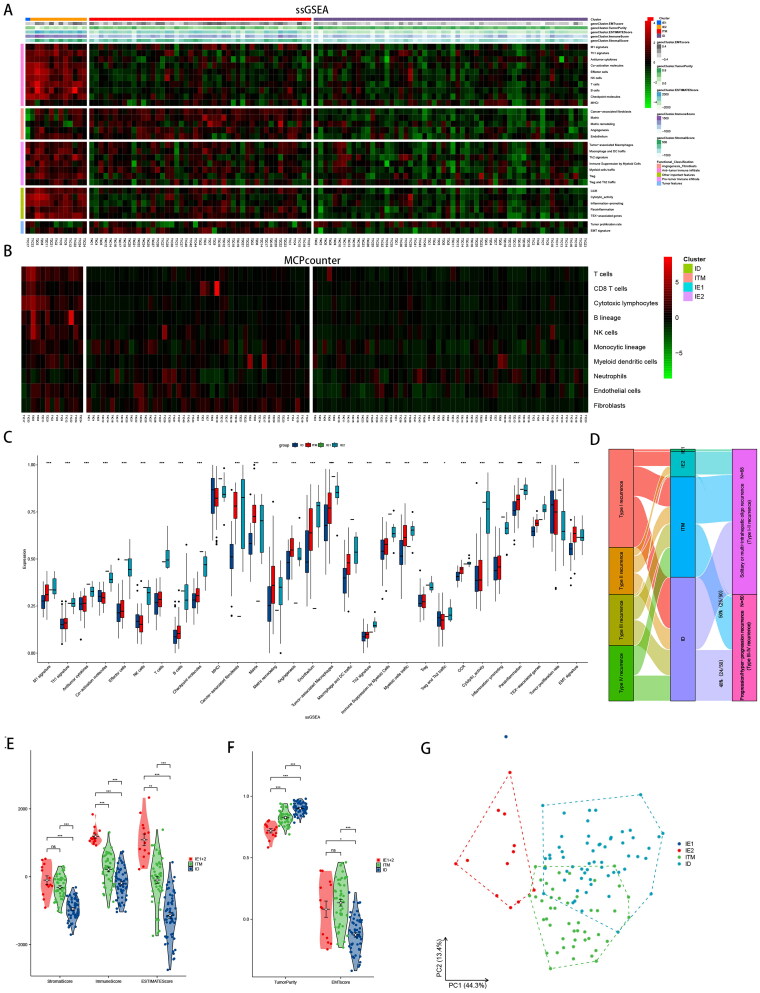
**The four distinct tumour microenvironment (TME) subtypes identified in resectable hepatocellular carcinoma (HCC).** (A) Heatmap of normalized ssGSEA scores for EMT score, tumour purity, ESTIMATE score, immune score and stromal score. Tumour samples were grouped into four immune classes, IE1 (immune enriched/matrix-poor), IE2 (immune enriched/matrix-rich), ITM (immune intermediate/matrix-rich) and ID (immune desert/matrix-poor) based on 29 Fges. (B) Heatmap for immune responses based on the MCP-COUNTER algorithms in the four immune classes. (C) The violin plots revealed that the immune cell types were differentially expressed in the four immune classes via ssGSEA. (D) The flow among recurrence types and immunophenotyping. (E) Violin plot analysis comparing the ESTIMATE score, stromal score and immune score among IE1 and IE2, ITM and ID (*t*-test). (F) Violin plot analysis comparing the tumour purity and EMT score among IE1 and IE2, ITM and ID (*t*-test). (G) Four immune classes PCA.

The infiltration levels of the 29 immune cells in each HCC sample were determined using ssGSEA. Patients were stratified into low (*n* = 105) and high (*n* = 13) immune groups (Figure S1(A,B)). The high immune groups were divided into immune enriched/matrix-poor (IE1, *n* = 1) and immune enriched/matrix-rich (IE2, *n* = 12) groups based on fibrosis (Figure S1(C,D)). The low immune groups were divided into immune intermediate/matrix-rich (ITM, *n* = 47) and immune desert/matrix-poor (ID, *n* = 58) groups (Figure S1(E,F), Figure 2(A)). Immune enrichment in recurrent HCC was primarily stroma-rich, whereas III and IV recurrences were mainly contained in the ITM and ID clusters (Figure 2(A–D)). Most immune cells had extremely high levels in the IE clusters compared to that in the ITM and ID clusters, whereas the ID cluster had extremely poor levels of immune cells and stromal infiltration. The immune characteristics of the ITM clusters were intermediate between those of the IE and ID clusters. These results were verified using MCP counter analysis (Figure 2(B)).

The proportions of T cells, B cells, NK cells and Tregs were markedly higher in the IE cluster than in the ITM and ID clusters, with the matrix composition in agreement (Figure 2(C)), highlighting the reliability of ssGSEA-based immune clusters.

All three scores of the ITM and ID clusters were lower than those of the IE cluster (*p* < .05) (Figure 2(E)), consistent with the ssGSEA results. This result also reflects the low level of tumour purity in the ITM and ID clusters (Figure 2(F)). Additionally, based on the PCA of bulk RNA-seq datasets using ssGSEA results, IE clusters were clearly distinguished from ITM or ID clusters, with ITM clusters slightly intersecting with ID features (Figure 2(G)). Given that ITM represents an intermediate transition state between IE and ID, serving as an indicator of crossover prognosis, it is reasonable to expect a characteristic crossover between ITM and ID.

### Weighted gene co-expression network analysis mining with ITM and type ID co-expressed gene sets

**Figure 3.**
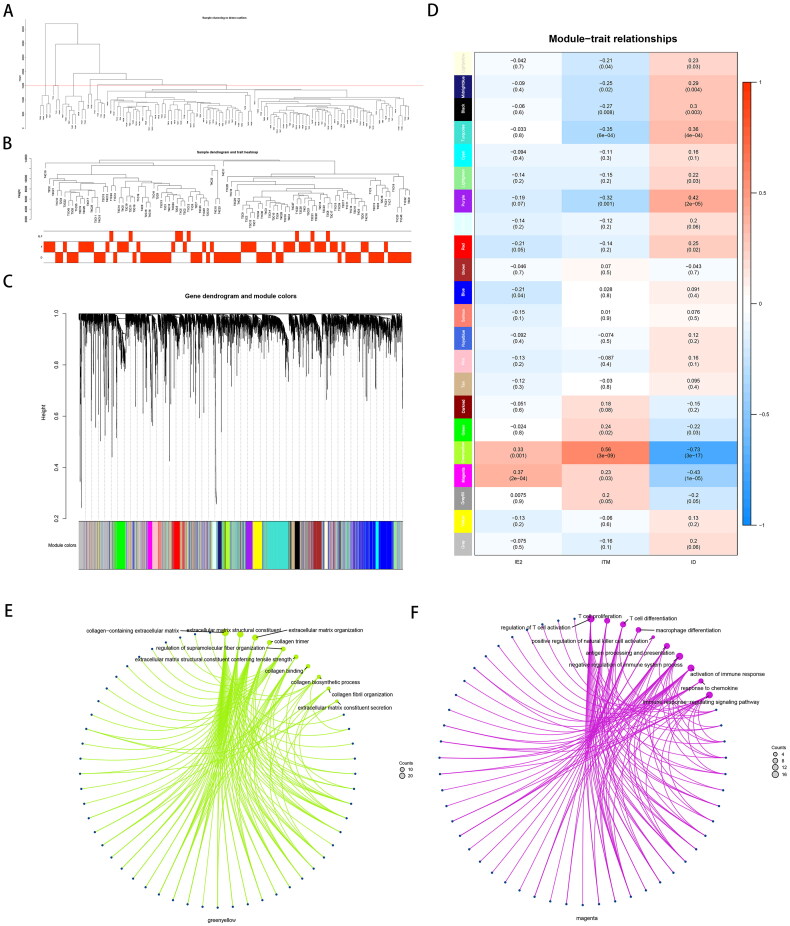
**Weighted gene co-expression network analysis (WGCNA) results in different immune subtypes of resectable HCC.** (A, B) Sample dendrogram. (C) Sample dendrogram and trait heatmap. (D) Module–trait relationships: every module has its correlation coefficient and corresponding *p* value. The results show that the green–yellow module and the magenta module are significantly and strongly correlated with immune clustering. (E) Functional enrichment analysis of co-expressed genes in the magenta module suggested that they were mainly immune-related. (F) Functional enrichment analysis of co-expressed genes in the green–yellow module suggested that they were mainly matrix related.

Of the 118 HCC samples analysed, 95 met the criterion of *h* < 15,000 and were selected for WGCNA to identify crucial module genes involved in the immunophenotyping of recurrent HCC (Figure 3(A)). Sample correlation with immunophenotyping and module clustering of co-expressed gene sets (Figure 3(B)) were also demonstrated. The smallest value of *β* (i.e. six; Figure S2(A,B)) was selected, leading to a nearly scale-free network with a truncated scale-free fitting index of R2 = 0.90 (Figure S2(C,D)). Two modules showed a significant correlation with immunophenotyping, namely, the green–yellow and magenta modules, which were significantly positively correlated with the IE and ITM clusters and significantly negatively correlated with the ID cluster (Figure 3(D)). The correlation between the magenta module and the ITM was significant but weak (*p* = .03, cor = 0.23), reflecting the characteristics of the immune transition state. The 159 genes in the magenta module were primarily associated with immune cell activation and function (Figure 3(E)). Similarly, 138 genes in the green–yellow module were primarily associated with the construction of the matrix environment (Figure 3(F)). Additionally, further differential gene expression analysis and functional enrichment in the ITM group comparing primary tumours of patients with type III–IV recurrence versus type I–II recurrence revealed a significant downregulation of genes associated with type III collagen, which is closely linked to tumour dormancy (Figure S3).

### Analysis of target module protein interactions and drug regulatory networks

**Figure 4.**
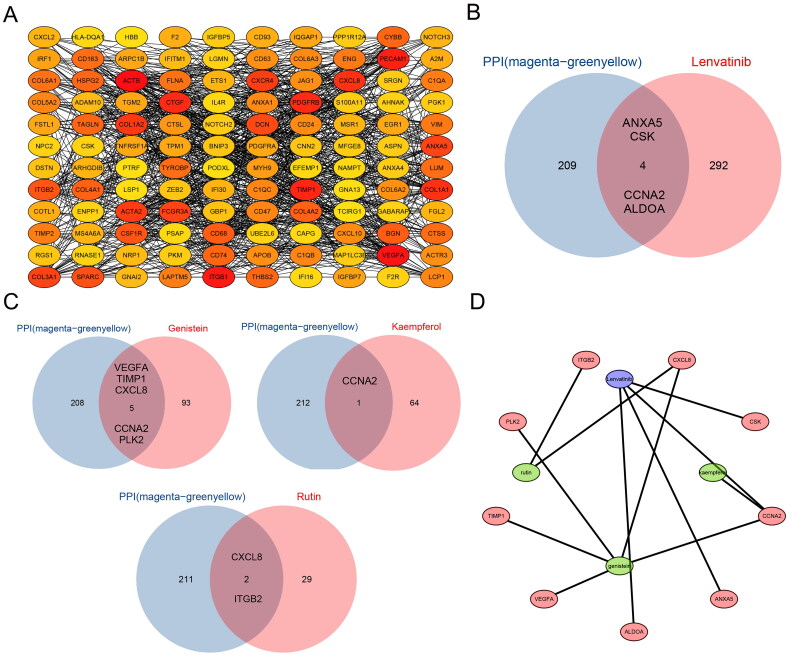
**Analysis of protein–protein interaction network and drug target regulatory network of genes in green–yellow and magenta modules.** (A) Key networks and highly interconnected genes were identified using CytoHubba (top 120). (B) The intersection of highly correlated genes with lenvatinib therapeutic targets. (C) The intersection of highly correlated genes with therapeutic targets of main components of Huaier granules (HG). (D) Regulatory network of intersection targets and major components of HG and lenvatinib.

Using the STRING database, we constructed a protein–protein interaction network comprising 297 genes from the two identified modules. The network was visualized in Cytoscape, and hub genes were ranked using CytoHubba, with the top 120 genes displayed in Figure 4(A). Subsequently, the intersection of lenvatinib targets and key genes yielded four matching genes (ANXA5, CSK, CCNA2 and ALDOA) (Figure 4(B)). In contrast, the intersection of the key genes and the four active component targets of HG (genistein, glucuronic acid, kaempferol and rutin) yielded six matching genes (VEGFA, TIMP1, CXCL8, CCNA2, PLK2 and ITGB2), which align with the targets of genistein, kaempferol and rutin (Figure 4(C)). The regulatory network of the matched targets with the active components of each drug is shown in Figure 4(D). A list of drug targets is presented in Table S2.

### Validation of expression of each potential target in single-cell nuclear sequencing

**Figure 5.**
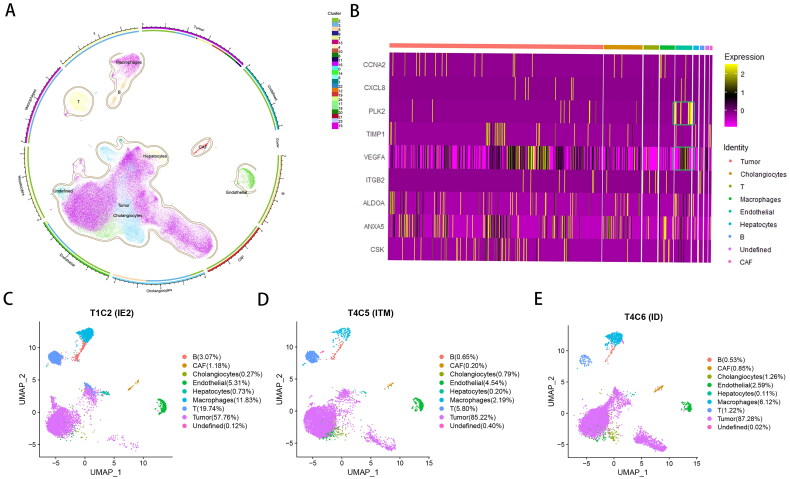
**Single-cell nuclear sequencing analysis validates the expression of potential therapeutic targets.** (A) Cell type landscape of tumour samples from 11 HCC patients. (B) Expression of nine potential therapeutic targets in various cell types. (C–E) Representative display of cell types from primary tumours of patients with different immune classes (C: IE2; D: ITM; E: ID).

Figure 5 illustrates the single-cell level expression profiles of potential therapeutic targets. Figure 5(A) provides an overview of the cellular composition at the single-cell level in primary tumours from 11 patients. Figure 5(B) shows that the nine potential drug targets were expressed in multiple cell types, including tumour cells and the expression of the markers PLK2 and VEGFA was particularly prominent in endothelial cells. Additionally, the cell type distribution of representative samples in each immune group is shown in Figure 5(C–E).

### Drug-target molecular docking simulation

**Figure 6.**
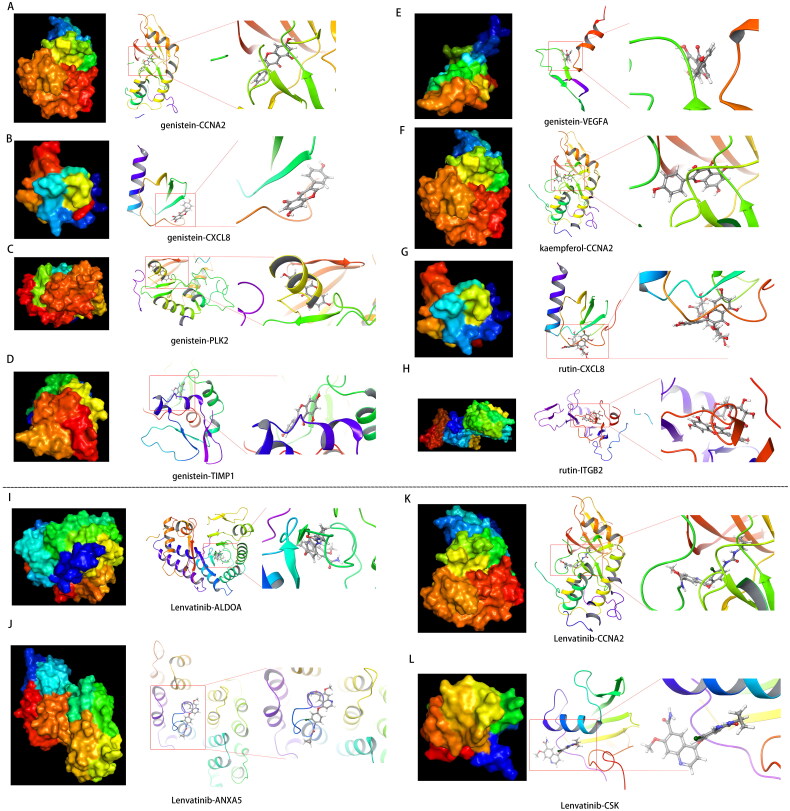
**Drug-target molecular docking simulation.** (A–H) Schematic diagram of the docking of Huaier active ingredients and potential therapeutic targets (A: genistein-CCNA2; B: genistein-CXCL8; C: genistein-PLK2; D: genistein-TIMP1; E: genistein-VEGFA; F: kaempferol-CCNA2; G: rutin-CXCL8; H: rutin-lTGB2). (I–L) Schematic diagram of the docking of lenvatinib active ingredients and potential therapeutic targets (I: lenvatinib-ALDOA; J: lenvatinib-ANXA5; K: lenvatinib-CCNA2; L: lenvatinib-CSK).

A molecular docking analysis was performed using the Schrodinger Maestro program to assess the binding affinity of the potential drugs to their targets. The ligands were docked to the active sites of the protein targets, and each drug candidate formed discernible hydrogen bonds with its target and exhibited strong electrostatic interactions (Figure 6 and Figure S4). Furthermore, the docking scores revealed highly stable binding for each drug–target pair, with nearly all combinations achieving a Glide Score below −5 kcal/mol (Figure S4).

### Verification of the expression of VEGAF and PLK2 in primary tumours of type III–IV patients by immunohistochemistry

**Figure 7.**
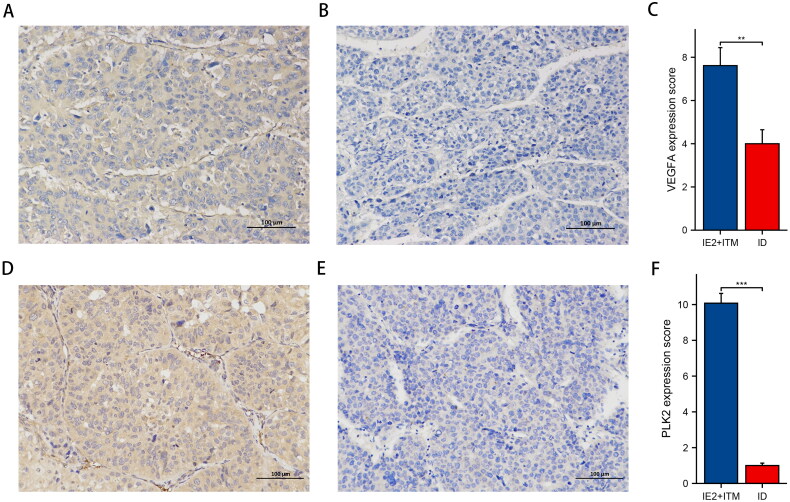
**Validation of VEGFA and PLK2 expression in III–IV recurrent hepatocellular carcinoma (HCC).** (A) Expression of VEGFA protein in cancer tissues of ITM type HCC patients. (B) Expression of VEGFA protein in cancer tissues of ID type HCC patients. (C) Comparison of VEGFA protein expression in cancer tissues of ITM type (*n* = 25) and IE2 type (*n* = 1) HCC patients and ID type (*n* = 24) patients. (D) Expression of PLK2 protein in cancer tissues of ITM type HCC patients. (E) Expression of PLK2 protein in cancer tissues of ID type HCC patients. (F) Comparison of PKL2 protein expression in cancer tissues of ITM type (*n* = 25) and IE2 type (*n* = 1) HCC patients and ID type (*n* = 24) patients. **p < .01 and ***p < .001.

VEGAF and PLK2 are representative markers associated with ITM in the primary tumours of patients with type III–IV recurrence. The visibility of tissue sections was evident, with a notable increase in VEGFA and PLK2 expression in ITM (*n* = 25) and IE2 (*n* = 1) cancer tissues compared to the ID group (*n* = 24) (Figure 7).

## Discussion

ICB therapies have emerged as promising immunotherapeutic approaches for HCC [38,39]; however, their response rate remains limited owing to adaptive resistance or primary mechanisms [40]. Type III–IV recurrence primarily involves patients with vascular invasion, extrahepatic metastasis and multifocal recurrence [3], indicating that the unique immune microenvironment supports micro-tumorous foci invasion and proliferation. Consequently, TME is strongly associated with the postoperative recurrence of HCC. This study addresses a crucial gap by focusing on the TME and the therapeutic potential of ICB in resectable HCC, particularly in cases of type III–IV recurrence. A comparative analysis of patients who experienced recurrence following radical HCC treatment indicated that postoperative primary tumours associated with type III–IV recurrence exhibited markedly immunosuppressive characteristics.

This study did not rely solely on unsupervised analyses to mitigate any potential bias introduced by machine learning algorithms. Instead, progressive unsupervised immunotyping of samples was performed by incorporating a previous theoretical basis, a key strength of the study. Three primary cancer immune phenotypes exist: ID, immune-excluded and inflamed [21]. Immune deserts are characterized by low immune cell infiltration, a lack of adequate T cell activation, and immunological ignorance. In contrast, inflamed tumours exhibit immune cell infiltration and CAF enrichment. Immunologically excluded tumours may reflect the presence of specific vascular factors, barriers or stroma-based suppression, representing an intermediate state between immune deserts and immune inflammation. Based on this foundation, Bagaev et al. proposed four immune subtypes, further dividing immune inflammation into IE (fibrotic) and IE (non-fibrotic), whereas IDs were subdivided into fibrosis and desert subtypes based on the extent of fibrosis [22]. In the current study, two primary features of mainstream immunotyping (immune cell infiltration and matrix enrichment) were extracted, and unsupervised analysis of the samples identified four unique subtypes, namely IE1, IE2, ITM and ID, in patients with recurrent HCC. The proportion of patients with IE1 was extremely low, and patients with HCC recurrence were primarily categorized into three subtypes: IE2, ITM and ID. This distribution likely reflects the varying degrees of liver fibrosis associated with HCC. Although the immune microenvironment did not appear to be a decisive factor in malignant recurrence behaviour, patients with type III–IV relapse were predominantly categorized into the ITM and ID subtypes, consistent with previous findings. In ID-type primary tumours, tumour purity was high, whereas immune infiltration and matrix enrichment were low, implying a limited capacity to stimulate immune cells through ICB therapy. Low-dose radiotherapy has been suggested as a potential strategy to counteract immune desertification in such cases [41].

Encouragingly, for patients with ITM-type HCC, targeting stromal-associated components offers a potential strategy to mitigate the immunosuppressive state and enhance the efficacy of ICB therapy. The crosstalk between matrix components – including CAFs, endothelial cells and ECM – and immune cells represents an intermediate transition state between immune enrichment and desertification. This state is closely associated with tumour proliferation, invasion and drug resistance [42]. CAFs, endothelial cells and their secreted factors are highly involved in ECM packing and remodelling [43–46]. The ECM contributes to tumour immunosuppression by inhibiting tumour cell death and interfering with tumour antigen presentation, recognition, activation, migration and the killing activities of effector T cells [47–49]. Tumour types with poor responses to ICB therapy, such as pancreatic, breast and colorectal cancers, are characterized by collagen-dense ECM [50]. This phenomenon also applies to HCC, as patients frequently exhibit hepatic fibrosis. Therefore, ITM patients with HCC thus represent the most suitable population for reversing immune rejection using tumour-associated stroma inhibitors or ECM-targeting agents. These approaches hold significant potential for guiding precise postoperative anti-recurrence therapies in patients with HCC.

Another interesting finding is the difference in HCC prognosis in the ITM cluster. Differential analysis and pathway enrichment were performed on the expression profiles of patients with type III–IV and type I–II tumours in this group. The collagen-containing ECM pathway in primary tumours of patients with type III–IV was significantly downregulated, with many genes related to type III collagen showing significant downregulation. Type III collagen prevents cancer cells from reawakening by inducing and maintaining dormancy at the primary site [51]. This observation may explain the differences in the prognosis for patients in the ITM cluster.

In this study, co-expressed gene sets that exhibited a significant positive correlation with ITM were selected for protein interaction analysis, and their key network and highly interconnected genes were identified. Several of these genes matched the targets of lenvatinib and HG, and a drug–target model was successfully constructed using molecular docking. This finding suggests that lenvatinib and HG may be effective choices for managing postoperative recurrence in patients with ITM-type HCC.

Lenvatinib is an oral small molecule that inhibits multiple receptor tyrosine kinases [31]. This study indicated that ALDOA, CCNA2, ANXA5 and CSK – which are key genes related to ITM – are lenvatinib targets, highlighting its potential use in postoperative anti-relapse therapy for ITM patients with HCC. However, existing studies and clinical observations have shown that, despite an initial response, most patients eventually develop lenvatinib resistance and disease progression, thereby limiting its efficacy and application [31,52]. Resistance mechanisms include the activation of EGFR and stimulation of the EGFR–STAT3–ABCB1 axis, suggesting that EGFR inhibitors may be effective in addressing acquired lenvatinib resistance in HCC [53]. However, side effects of lenvatinib include proteinuria, hypothyroidism [54] and hypertension [55].

Given the limitations of lenvatinib, the focus was shifted to traditional Chinese medicine extract HG, an established antitumor agent with antiproliferative, anti-metastatic and antiangiogenic properties. HG induces apoptosis, reverses multidrug resistance, inhibits tumour stem cells and modulates tumour-specific immunity [56–58]. It is a multipronged, comprehensive antitumor drug with low toxicity [58,59], comprising various organic ingredients and over 10 types of minerals. Active ingredients such as genistein, glucuronic acid, kaempferol and rutin target multiple pathways [36,56]. This study showed that multiple crucial genes (VEGFA, TIMP1, CXCL8, CCNA2 and PLK2) related to ITM align with genistein, kaempferol and rutin targets of HG. These targets are closely related to CAFs (TIMP1 and CXCL8), angiogenesis (VEGFA) and cell proliferation (CCNA2 and PLK2); hence, HG is a promising antitumor-promoting stromal inhibitor to reverse the immune rejection state of TIM. Undoubtedly, the role of HG in antitumor therapy for TIM-type HCC is significant. Notably, while EGFR was not identified as a key ITM-associated factor, it was found in the target region of genistein, suggesting that HG may have dual inhibitory effects on EGFR and VEGF. This finding supports the hypothesis that combining HG with lenvatinib could overcome lenvatinib resistance. Notably, some studies have explored the inhibitory effects of other functional molecules on targets such as PLK2 [60,61]. However, these single molecules are still in the early experimental stages, whereas HG has been extensively clinically validated as a standardized pharmaceutical product [62].

This study has several significant implications. By selecting patients who experienced recurrence after radical HCC resection and performing immune grouping, advancements in precise immunotherapy were explored, thereby addressing the gap in research on the TME of resectable HCC, especially in the context of recurrence. ITM was identified as the primary target population of ICB, and two promising drugs, lenvatinib and HG, were proposed to reverse the tumour immune rejection status, supported by virtual docking validation. Notably, type III–IV recurrence typically occurs within 2 years after surgery, and most type IV recurrences occur within half a year after surgery. These observations highlight the narrow intervention window for postoperative anti-recurrence therapy in high-risk patients, particularly type IV cases. Therefore, the combination of lenvatinib or HG with ICB may be an adjuvant worth considering for patients at high risk of type III–IV recurrence. These findings have considerable value in advancing anti-recurrence therapy after resectable HCC and in supplementing systemic treatment strategies for HCC.

Furthermore, this study provides a foundation for integrating immunotherapy with precision medicine in HCC management. By identifying immune subtypes and exploring targeted therapies based on immune phenotypes, the findings provide a foundation for personalized treatment approaches. The integration of ICB with stromal-targeting agents could significantly improve outcomes for patients, particularly those with high-risk recurrences, bridging the gap between systemic treatments and precise, individualized therapies. As immunotherapy and precision oncology continue to evolve, these advancements have the potential to transform current treatment paradigms for HCC.

However, our study has some limitations. First, given the limitations of our retrospective study design, the sample size was relatively small, and all included patients were from a single centre, which may introduce selection bias. Therefore, it is imperative to undertake a comprehensive, multicentre prospective validation in order to bolster the findings. Second, further experimentation is necessary in subsequent research endeavours to authenticate the intricate data analysis and virtual molecular docking utilized in this study.

In conclusion, the immunophenotypes observed in recurrent HCC were primarily characterized by IE2, ITM and ID, and type III–IV recurrences were dominated by ITM and ID. Nine molecules, including VEGFA and PLK2, were promising targets for potential inhibitors capable of reversing the immune intermediate state in HCC. Additionally, HG and lenvatinib showed promise as adjuvant treatment options for patients with immune intermediates and can be used as multitarget ECM inhibitors with ICB treatment under certain conditions, particularly for type III–IV recurrence. However, a comprehensive, multicentre prospective validation is necessary to substantiate these findings. Additionally, further experimental studies are needed to validate the intricate data analysis and molecular docking results.

## Supplementary Material

Supplementary_Material - IANN-2024-0003.R1.docx

## Data Availability

All data needed to evaluate the conclusions in the paper are present in the paper and/or the Supplementary Materials. Additional data related to this paper may be requested from the authors (Lu-Nan Qi qilunan_gxmu@163.com, wangzhennuo@aliyun.com; Yu-Chong Peng pengyuchong2018@126.com). For any questions regarding the data used in this study, please contact the authors.
