# Supplementary material for: Immune infiltration landscape and potential drug-targeted implications for hepatocellular carcinoma with ‘progression/hyper-progression’ recurrence
Source: Ann Med. 2025 Jan 27;57(1):2456113. doi: 10.1080/07853890.2025.2456113 (PMC11774162; doi:10.1080/07853890.2025.2456113)
Supplement: Supplementary_Material - IANN-2024-0003.R1.docx [file IANN_A_2456113_SM0604.docx]

***Supplementary Material***

**Supplementary Figures and Tables (Fig S1-4, Table S1-2)**

**1.1 Supplementary Figures**


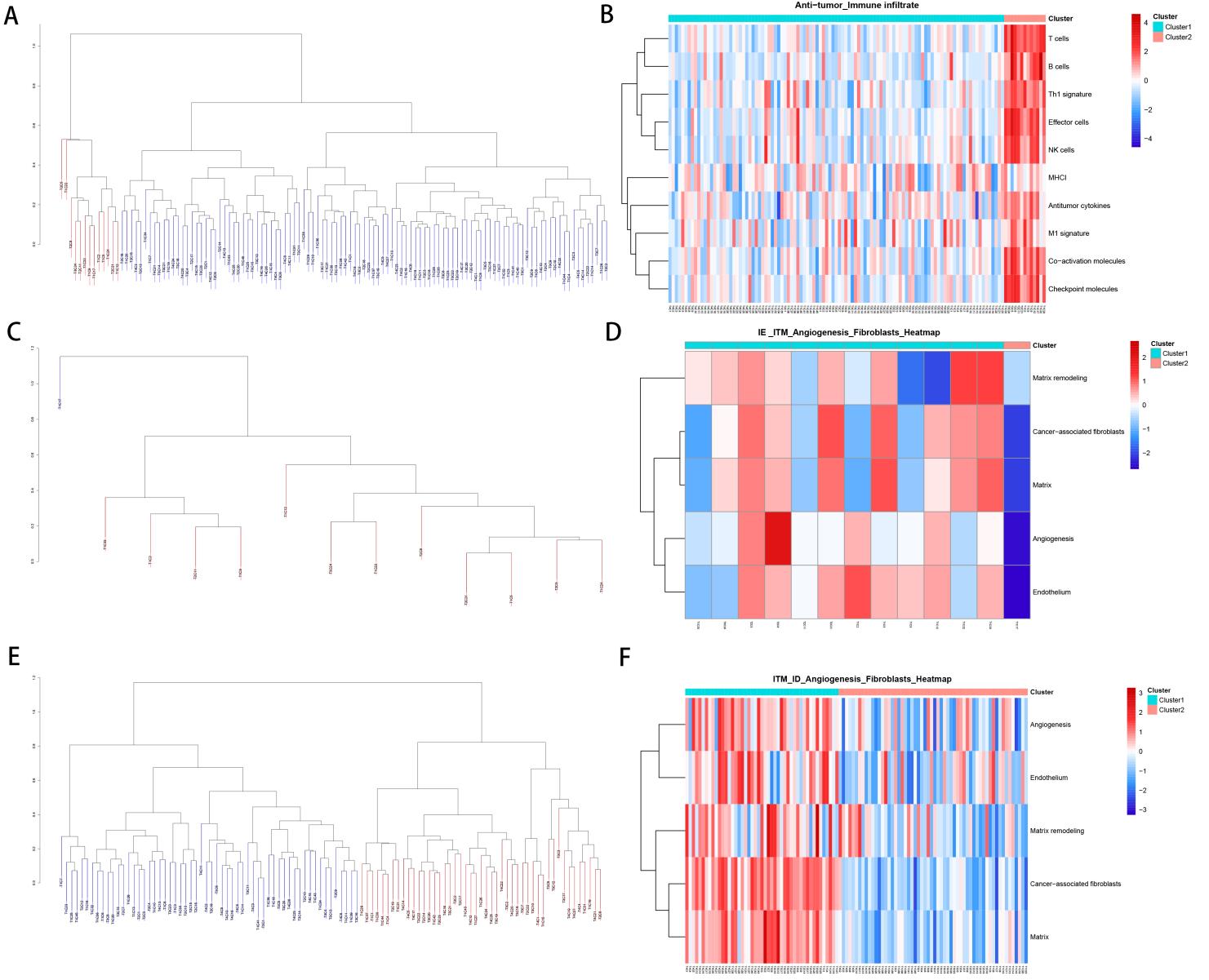


**Figure S1** Step-by-step unsupervised immune infiltration analysis. (A-B) Unsupervised clustering analysis of all samples based on ssGSEA score of 24 immune celltypes. Unsupervised clustering of all samples based on the ssGSEA score for functional classificationof anti-tumor immune infiltration into high (red) and low (blue) immune groups. (C-D) Unsupervisedclustering of high immune group samples based on the ssGSEA score of the angiogenesis fibroblastclassification into lEl (blue, immune-enriched/matrix-poor, n=l) and lE2 (red, immune-enriched/matrix-rich, n=12) groups. (E-F) Unsupervised clustering of low immune group samples based onthe ssGSEA score ofthe angiogenesis fibroblast classification into ITM (red, immuneintermediate/matrix rich,n =47) and ID (blue, immune desert/stroma poor, n = 58) groups..

Supplementary Material


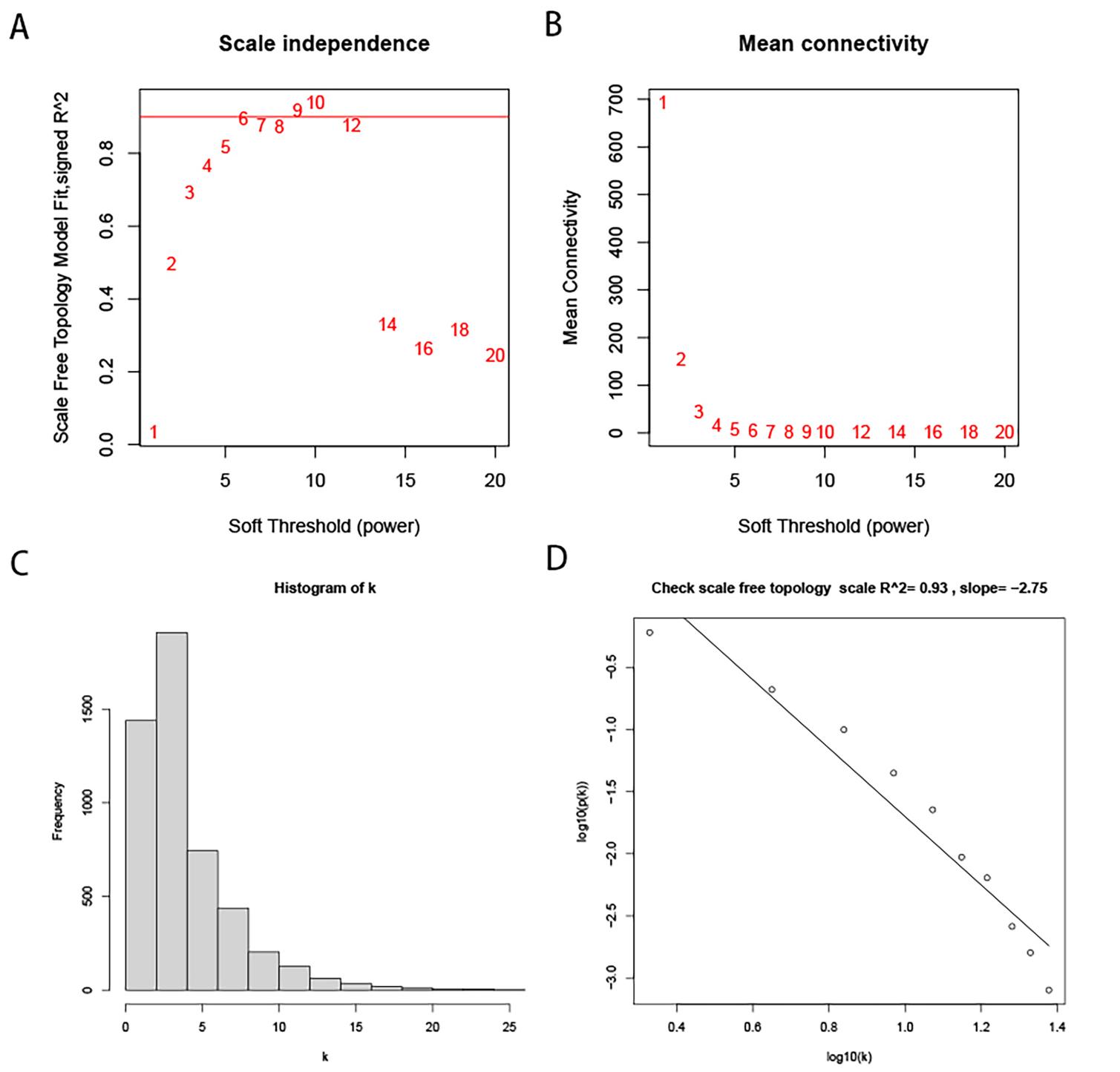


**Figure S2** Soft-threshold power β selection. (A) Analysis of scale-free fit index for various soft- threshold powers, the red line is set at 0.9: (B) An analysis of mean connectivity for various

softthreshold powers; (C) Histogram of k when β=6; (D) Checking scale-free topology with β=6.


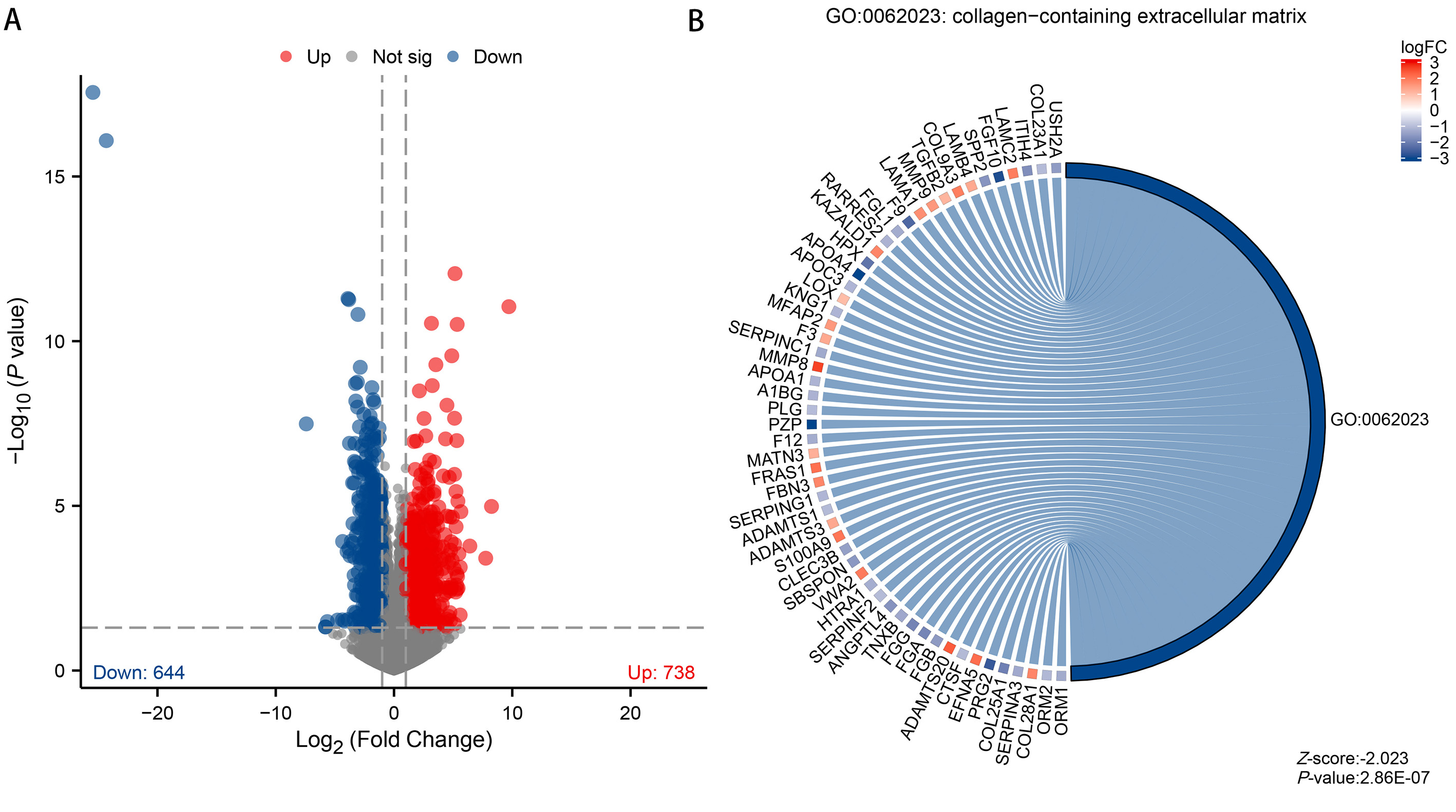


**Figure S3** Differentially expressed gene (DEG) analysis and enrichment analysis of gene expression profiles of Type III-IV and TypeI-II primary tumors in ITM group. (A) The volcano plot shows the expression profiles of 1382 DEGs in HCC between type I-II and type III-IV. The high-expressing

genes (n=738) are shown in red and the low-expressing genes (n=644) are shown in blue. (B) Gene Ontology (GO) Enrichment Analysis reflects that the collagen-containing extracellular matrix

pathway was significantly down-regulated and the genes related to type III collagen were significantly down-regulated.


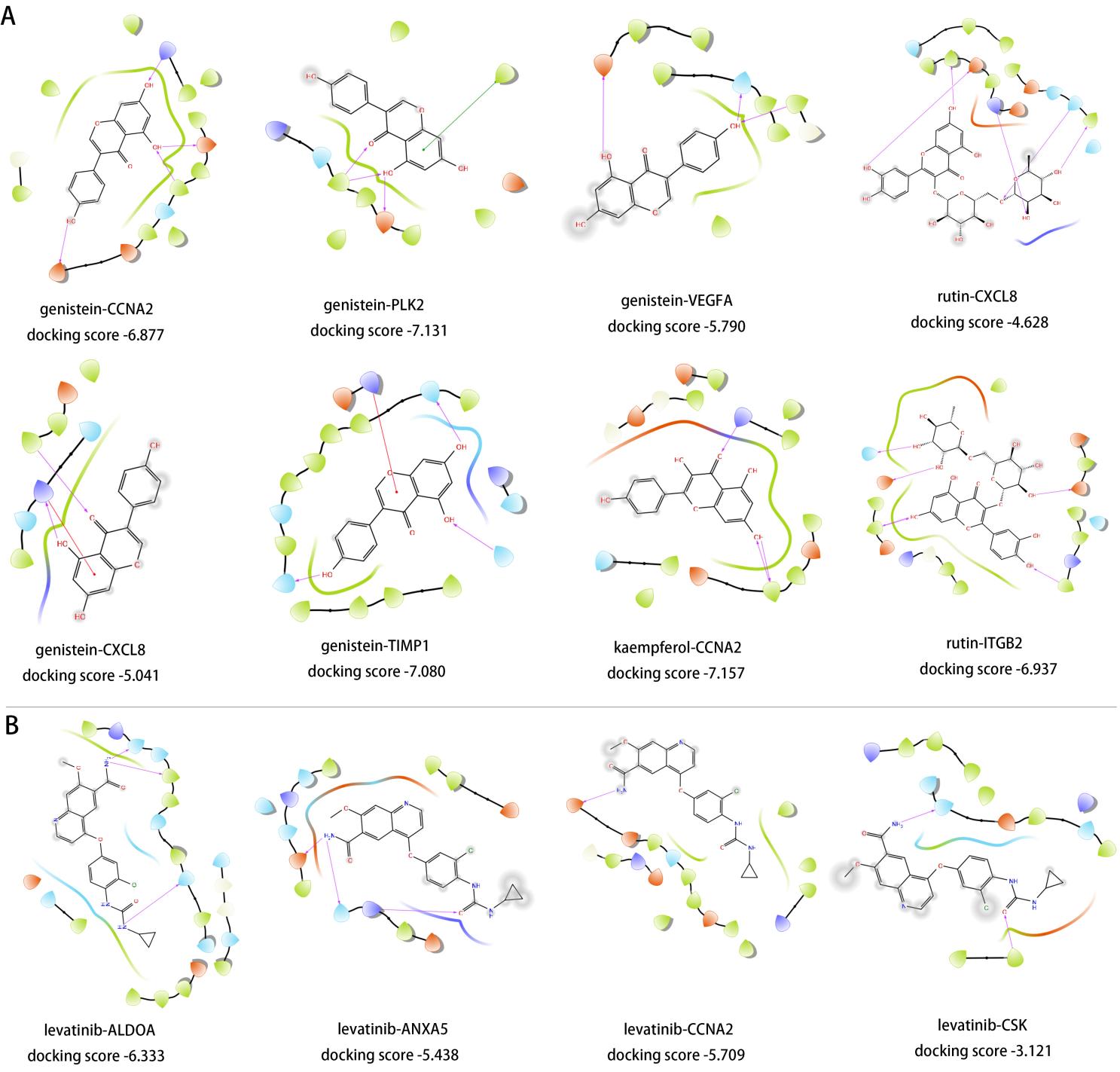


**Figure S4** Drug-target molecular docking simulation (2D). (A) Schematic diagram of the docking of Huaier active ingredients and potential therapeutic targets. (B) Schematic diagram of the docking of lenvatinib active ingredients and potential therapeutic targets.

**1.2 Supplementary Tables**

**Table S1 Partial KEGG/GO pathway enrichment results (type III-IV recurrence VS type I-II recurrence)**

| ID | Description | P.value | P.adjust | Q.value | geneID | Count | zscore |
| --- | --- | --- | --- | --- | --- | --- | --- |
| GO:0002717 | positive regulation of natural killer cell  mediated immunity regulation of natural killer cell mediated | 0.000166982 | 0.005622114 | 0.004830661 | CLNK/IL12B/CD160/IL21/KLRC4/SH2 D1B/KLRC2/KLRK1  CLNK/IL12B/CD160/IL21/LEP/KLRC4 | 8 | -2.828427125 |
| GO:0002715 | immunity  humoral immune | 0.000195747 | 0.006287089 | 0.005402024 | /SH2D1B/KLRC2/KLRK1/HLA-B  MASP2/LTF/C8B/C6/HPX/C7/C9/TRE M1/JCHAIN/SPINK5/CFHR5/FCN3/C  XCL13/C8A/AIRE/FCN2/LEAP2/DEFB 1/PLA2G1B/FGA/FGB/REG1B/COLEC 10/DMBT1/PAX5/GALP/BPIFA1/IGH | 10 | -2.529822128 |
| GO:0006959 | response  natural killer cell | 0.003915534 | 0.054189672 | 0.046561125 | D  CLNK/IL12B/CD160/IL21/LEP/KLRC4 | 28 | -3.401680257 |
| GO:0002228 | mediated immunity  myeloid cell activation involved in immune | 0.00495879 | 0.063707399 | 0.054738995 | /SH2D1B/KLRC2/KLRK1/HLA-B  PTGDS/CLNK/BLK/ANXA3/CPLX2/E NPP3/DNASE1L3/PTGDR/ADORA2B/ | 10 | -2.529822128 |
| GO:0002275 | response | 0.008192429 | 0.089877392 | 0.07722491 | CD177/FER1L5  FOXP3/CLNK/HPX/IL12B/CD160/PCK 1/SPINK5/BLK/IRF4/IL21/FCN3/ENPP 3/FCN2/FCRL3/INAVA/DNASE1L3/A | 11 | -0.301511345 |
| GO:0002697 | regulation of immune effector process | 0.009075289 | 0.098021155 | 0.084222234 | DORA2B/MZB1/CCL19/LEP/ZNF683/ CLEC4G/KLRC4/COLEC10/SH2D1B/C D177/KLRC2/KLRK1/HLA-B  FOXP3/CLNK/HPX/IL12B/CD160/PCK 1/BLK/IL21/FCN3/ENPP3/FCN2/INAV | 29 | -3.528211425 |
| GO:0002699 | positive regulation of immune effector  process  CCR chemokine | 0.009337837 | 0.099950101 | 0.085879634 | A/ADORA2B/MZB1/CCL19/KLRC4/C OLEC10/SH2D1B/CD177/KLRC2/KLR K1/HLA-B  CCL7/CCL20/CCL25/CCL21/XCL2/CX | 22 | -3.411211462 |
| GO:0048020 | receptor binding  chemokine receptor | 0.002743311 | 0.03588077 | 0.030297891 | CL13/DEFB1/CCL19  CCL7/CCL20/CCL25/CCL21/XCL2/CX | 8 | -1.414213562 |
| GO:0042379 | binding | 0.011293536 | 0.097956567 | 0.082714986 | CL13/DEFB1/CCL19/S100A14 | 9 | -1 |

WGCNA_PPI

Degre e

Gene

Lenvatinib_tar get

Huaier_target genistein glucuronic acid kaempferol rutin

| ACTB | 84 | CAH2 | AKT1 | SOD1 | ACHE | SIRT1 |
| --- | --- | --- | --- | --- | --- | --- |
| VEGFA | 59 | ANDR | AKR1B1 | GRIA2 | AHR | TFAM |
| ITGB1 | 47 | P49137 | BIRC5 | CA2 | AKT1 | UCP1 |
| PECAM1 | 41 | ESR1 | APOA1 | NOS2 | ALOX5 | PPARGC1A |
| TIMP1 | 38 | TTHY | KLK3 | LCT | AR | CXCL1 |
| PDGFRB | 37 | CCNA2 | AR | DPEP1 | BAX | S100A8 |
| CTGF | 37 | KIF11 | ATM | PTGS2 | BCL2 | STAT4 |
| CXCL8 | 36 | P11142 | BCL2 | DPP4 | CALM3 | IFNG |
| COL1A1 | 36 | NONE | BAX |  | CASP3 | MPO |
| COL1A2 | 35 | P62937 | BTK |  | CHRM1 | ALOX5 |
| ACTA2 | 33 | O14965 | BUB1 |  | CYP1A1 | C5AR1 |
| CXCR4 | 33 | PIM1 | CASP3 |  | CYP1A2 | CASP3 |
| FCGR3A | 32 | PK3CG | CCNB1 |  | CYP1B1 | CAT |
| ANXA5 | 32 | CAH1 | CASP9 |  | CYP3A4 | DIO1 |
| COL3A1 | 32 | ALBU | CCNA2 |  | DIO1 | FCER2 |
| DCN | 31 | VTDB | CDC20 |  | ESR1 | GSTP1 |
| SPARC | 30 | MK10 | CDC25C |  | ESR2 | HAS2 |
| ITGB2 | 29 | EGFR | CDKN1A |  | GSTM1 | HMGCR |
| CSF1R | 29 | CFAB | CDK2 |  | GSTM2 | IL1B |
| CD68 | 28 | MK01 | CFTR |  | GSTP1 | IL6 |
| BGN | 28 | ALDR | CDKN2A |  | HAS2 | CXCL8 |
| COL4A1 | 27 | CASP7 | CRYAB |  | HMOX1 | INS |
| COL4A2 | 27 | PDE5A | CYP24A1 |  | ICAM1 | ITGB2 |
| TAGLN | 25 | P24941 | FN1 |  | IKBKB | NOS2 |
| CYBB | 25 | CHLE | ESR1 |  | INSR | POR |
| HSPG2 | 25 | ESR2 | ERBB2 |  | JUN | PRKCB |
| CD74 | 25 | EPHB4 | ESR2 |  | MMP1 | RELA |
| THBS2 | 25 | THRB | EGFR |  | NOS2 | SOD1 |
| CD163 | 24 | GSTP1 | GLB1 |  | PGR | TBXA2R |
| CTSS | 24 | ADA17 | GFAP |  | POLD1 | TBXAS1 |
| TYROBP | 24 | PDE4B | GCK |  | PPARG | TNF |
| LUM | 24 | PRGR | FOS |  | PPP3CA |  |
| COL6A1 | 23 | CASP3 | HIF1A |  | MAPK8 |  |
| FLNA | 23 | CATD | HMGCR |  | PRSS1 |  |
| VIM | 22 | P35968 | ICAM1 |  | PSMD3 |  |
| ENG | 22 | AOFB | HPGD |  | PTGS1 |  |
| TIMP2 | 21 | P00390 | CXCL8 |  | PTGS2 |  |
| LAPTM5 | 21 | EST1 | IL1B |  | RELA |  |
| C1QA | 21 | STS | IGF1R |  | SELE |  |

Supplementary Material

| LCP1 | 20 | CD5R1 | JUN | SLC2A4 |
| --- | --- | --- | --- | --- |
| COL5A2 | 20 | MMP3 | LDLR | SLC6A2 |
| CD47 | 20 | MK08 | MDM2 | SLPI |
| C1QC | 20 | AK1C3 | KCNJ11 | STAT1 |
| C1QB | 20 | CHK1 | INS | TNF |
| CD24 | 19 | APOA2 | NOS3 | VCAM1 |
| CXCL10 | 19 | P62508 | NOS2 | XDH |
| COL6A3 | 19 | PPARG | MMP9 | AKR1C3 |
| ANXA1 | 19 | DHB11 | PCOLCE | NR1I2 |
| APOB | 18 | MCR | POLD1 | NR1I3 |
| JAG1 | 18 | BACE1 | PPARA | AHSA1 |
| COL6A2 | 18 | P10828 | PML | F7 |
| CD63 | 18 | PNPH | PPARG | GABRA1 |
| ACTR3 | 18 | Q03181 | PLK1 | PIM1 |
| CTSL | 17 | P07900 | MAPK1 | MAPK14 |
| MYH9 | 16 | CATS | MAPK3 | CDK1 |
| IGFBP7 | 15 | P14061 | MAP2K5 | NCOA2 |
| IRF1 | 15 | PYRD | PRSS1 | DPEP1 |
| FSTL1 | 14 | CATL2 | PTEN | CDK2 |
| TPM1 | 14 | PDE4D | PTGS2 | ADRA1B |
| TNFRSF1A | 14 | P11511 | RASGRF2 | GSK3B |
| IQGAP1 | 14 | HYES | PTGFR | CHRM2 |
| PDGFRA | 14 | P16083 | PTGER2 | CCNA2 |
| EGR1 | 14 | DYR | PTGS1 | CA2 |
| TGM2 | 14 | DHI1 | RELA | CHEK1 |
| ARPC1B | 14 | NOS3 | RAF1 | DPP4 |
| COTL1 | 13 | NQO1 | MAPK12 |  |
| CD93 | 13 | Q16539 | CCL2 |  |
| A2M | 13 | Q13231 | SELE |  |
| F2 | 13 | SHBG | SAFB |  |
| ARHGDIB | 12 | MMP13 | STAT3 |  |
| IFI30 | 12 | ANXA5 | SYK |  |
| FGL2 | 12 | RORA | TGFB1 |  |
| MSR1 | 12 | SRC | SOD2 |  |
| MS4A6A | 12 | O15496 | STAT1 |  |
| ETS1 | 12 | CFAD | TGFB2 |  |
| CXCL2 | 12 | FGFR1 | SULT1E1 |  |
| NOTCH3 | 12 | P08263 | TEP1 |  |
| GNAI2 | 11 | Q13126 | VCAM1 |  |
| IFITM1 | 11 | ZA2G | TP53 |  |
| ADAM10 | 11 | ANGI | TNF |  |
| NRP1 | 11 | P00517 | TIMP1 |  |
| DSTN | 10 | P63316 | VEGFA |  |

| GBP1 | 10 | Q06520 | NCOR1 |
| --- | --- | --- | --- |
| ZEB2 | 9 | MIF | MSLN |
| CNN2 | 9 | PH4H | HDAC6 |
| RGS1 | 9 | AMPM2 | MDC1 |
| BNIP3 | 9 | O15382 | AHSA1 |
| IGFBP5 | 8 | PDPK1 | PLK2 |
| ANXA4 | 8 | FA10 | UGT1A1 |
| SRGN | 8 | BMP7 | PIM1 |
| ASPN | 8 | LKHA4 | CHEK2 |
| MAP1LC3B | 8 | MMP8 | CA2 |
| PGK1 | 8 | P68400 | PBK |
| PPP1R12A | 8 | FKB1A | MAPK14 |
| PKM | 8 | FABP4 | CCNB1IP1 |
| NOTCH2 | 8 | O76054 | CHEK1 |
| MFGE8 | 8 | CATK | CDK1 |
| S100A11 | 8 | TYSY | CDC37 |
| AHNAK | 7 | CBR1 |  |
| RNASE1 | 7 | KC1G2 |  |
| NPC2 | 7 | QPCT |  |
| ENPP1 | 7 | BRAF1 |  |
| LGMN | 7 | ERG7 |  |
| CSK | 7 | PTN1 |  |
| UBE2L6 | 7 | P04745 |  |
| GABARAP | 7 | AKT1 |  |
| F2R | 7 | PLGF |  |
| EFEMP1 | 6 | DPP4 |  |
| IFI16 | 6 | RENI |  |
| PSAP | 6 | GSK3B |  |
| CAPG | 6 | P08069 |  |
| IL4R | 6 | PTN11 |  |
| NAMPT | 6 | P53350 |  |
| HLA-DQA1 | 6 | P04179 |  |
| PLSCR1 | 5 | MMP7 |  |
| LSP1 | 5 | HDAC8 |  |
| PODXL | 5 | SAHH |  |
| PTRF | 5 | ALDH2 |  |
| CCNA2 | 5 | Q08881 |  |
| CFLAR | 5 | ACK1 |  |
| GPNMB | 5 | FA7 |  |
| ITPR1 | 5 | TGFR1 |  |
| AKAP13 | 5 | KSYK |  |
| GM2A | 5 | RXRA |  |
| TCIRG1 | 5 | DCAM |  |
| GNA13 | 5 | O43617 |  |
| HBB | 5 | P09874 |  |
| CORO1B | 4 | DAPK1 |  |

Supplementary Material

| NFKBIZ | 4 | O00204 |
| --- | --- | --- |
| PSMB10 | 4 | MDM2 |
| SLC12A2 | 4 | UROK |
| TYMP | 4 | P21802 |
| C7 | 4 | ERBB4 |
| CLDN7 | 4 | FABP6 |
| PICALM | 4 | ADH1B |
| BTG2 | 4 | WASP |
| MT2A | 4 | Q04828 |
| IER2 | 4 | P16442 |
| RGS5 | 4 | P55263 |
| MPRIP | 4 | NR1H4 |
| ALDOA | 4 | PADI4 |
| JUNB | 4 | PNMT |
| VPS39 | 4 | ADHX |
| HBA1 | 4 | P14555 |
| CASP4 | 3 | P35558 |
| VMP1 | 3 | CALM |
| LGALS3BP | 3 | GALE |
| TCF4 | 3 | P00326 |
| ASAH1 | 3 | FNTA |
| GPX2 | 3 | DHSO |
| RNASET2 | 3 | CDK6 |
| LIMK2 | 3 | P30043 |
| MT1E | 3 | PDE3B |
| MT1G | 3 | MMP12 |
| MT1X | 3 | CTNA1 |
| CYFIP1 | 3 | P11474 |
| ANK3 | 3 | A1AT |
| TMSB4X | 3 | CDD |
| BMPR2 | 3 | LCK |
| SERPIND1 | 3 | P61586 |
| TNFAIP2 | 2 | P08581 |
| VPS13C | 2 | P78536 |
| LTBP3 | 2 | ISG20 |
| SEPT11 | 2 | HMDH |
| ENC1 | 2 | P54577 |
| ONECUT2 | 2 | TPH1 |
| PLK2 | 2 | PTGD2 |
| SH3BGRL | 2 | P15153 |
| TM4SF1 | 2 | FABP5 |
| LIMA1 | 2 | P22830 |
| DDIT4 | 2 | HXK1 |
| ENPP2 | 2 | AMYP |
| SLC4A7 | 2 | AK1C2 |
| PDE8A | 2 | P11586 |

| PLA2G12B | 2 | P01343 |
| --- | --- | --- |
| EVL | 2 | P12724 |
| A1CF | 2 | Q15382 |
| PANK3 | 2 | TYPH |
| PLTP | 2 | GLCM |
| RASGEF1B | 1 | P49638 |
| UACA | 1 | P06737 |
| MCFD2 | 1 | CSK |
| MGAT5 | 1 | IMDH2 |
| SPATS2L | 1 | P05451 |
| OSMR | 1 | P07195 |
| FAM129B | 1 | LGUL |
| RFX5 | 1 | P10827 |
| ELF3 | 1 | FABPH |
| ARHGAP21 | 1 | FABP7 |
| RARRES3 | 1 | PAK7 |
| LTBP4 | 1 | CCNT1 |
| NCALD | 1 | Q9NY01 |
| SEC14L1 | 1 | DCK |
| SULF2 | 1 | Q9HAN9 |
| SH3BGRL3 | 1 | GLYC |
| CD97 | 1 | ZAP70 |
| VEZT | 1 | Q14541 |
| TSFM | 1 | BST1 |
| CMTM6 | 1 | P34096 |
| MBNL1 | 1 | IMPA1 |
| ZFP36L2 | 1 | KPCT |
| CYBRD1 | 1 | BIRC7 |
| FRRS1 | 1 | TIE2 |
| TSPAN4 | 1 | JAK2 |
| SEPW1 | 1 | P32589 |
| MYO1D | 1 | P04745 |
| CCNI | 1 | P04183 |
| TMSB10 | 1 | HCK |
| SAT1 | 1 | ST1E1 |
| UBA3 | 1 | P49789 |
| ATP6V1B2 | 1 | PDK2 |
| WWTR1 | 1 | TGM3 |
| VPS8 | 1 | INSR  O00244 Q9BZX2 P17900  NR1H3 ITAL  O43252 IL2  CP2C9 |

Supplementary Material

P37243 NR1I3 RET4

PPARA AKT2

P46926 JAK3

ELNE

P61626

NR1H2

P05981

PPAP

GRB2

RAB5A

RB11A

Q07960

Q93088

MMP2

ALDOA GSTT2

GCR

NEP

CCL5

P29373

MMP9

P35520

PYR5

KTHY

P07602

S10A9

Q16775

P07686

Q9UBX1 CP2C8

DPEP1

B3GA1

Q9UKM7 FAK1

LEG2

MP2K1 LYAM3 SETD7 P20132 HXK4

ARSA NOS2

Q96CD2 P00519

NR1I2

VDR

P98170

TPIS

CD1A

G6PI

KIT

CATB

P07686

P19623

NGAL

P50613

OAT

PAK6

P03956

P08238

EPHA2 P00517

TGFB2

CLK1

RARG

SPYA

RASH

CMA1

ACADM C1S

GSTO1

Q9H4M7 ADA33

FA11
